# The role of minimal residual disease and serum free light chain ratio in the management of multiple myeloma

**DOI:** 10.1007/s12672-024-01090-1

**Published:** 2024-06-15

**Authors:** Long-Ying Zhu, Qi-Lei Hu, Liang Zhang, Zuo-Jie Li

**Affiliations:** 1 School of Medical Technology and Information Engineering, Zhejiang Chinese Medical University, Hangzhou, 310053 Zhejiang People’s Republic of China; 2 Department of Clinical Laboratory, First People’s Hospital of Linping District, Hangzhou, 311100 Zhejiang People’s Republic of China; 3 Department of Clinical Laboratory, The People’s Hospital of Cangnan Zhejiang, No. 2288 Yucang Road, Cangnan County, Wenzhou, Zhejiang 325800 People’s Republic of China

**Keywords:** Duration of Response (DOR), Minimal residual disease (MRD), Multiple myeloma (MM), Serum free light chain ratio (sFLCR), Stringent complete response (sCR)

## Abstract

Multiple myeloma (MM) denotes a cancerous growth characterized by abnormal proliferation of plasma cells. Growing evidence suggests that the complexity in addressing MM lies in the presence of minimal residual disease (MRD) within the body. MRD assessment is becoming increasingly important for risk assessment in patients with MM. Similarly, the levels of serum free protein light chain and their ratio play a crucial role in assessing the disease burden and changes in MM. In this paper, we review and explore the utilization of MRD and serum free light chain ratio in the treatment of MM, delving into their respective characteristics, advantages, disadvantages, and their interrelation.

## Introduction

Multiple myeloma (MM) ranks as the second most prevalent malignancy within the hematological system, marked by the malignant proliferation of B-lymphocytes that have undergone post-terminal differentiation. These cells originate either in the bone marrow or, less commonly, in extramedullary germinal centers, resulting in the production of immunoglobulins (IgG, IgA, IgD, and IgE immunoglobulins) and/or mono-light chains (κ/λ). Lonial S and Michels reported the treatment approaches for MM have seen ongoing enhancements and refinements, paralleling the rapid progress in medical technology [1, 2]. The integration of conventional medications along with innovative targeted drugs during autologous hematopoietic stem cell transplantation not only notably extends the duration of response (DOR) but also significantly lengthens overall survival (OS). While the inclusion or substitution of contemporary drugs in traditional treatment protocols markedly enhances the complete response (CR) rate, it is noteworthy that the CR rate for individuals with refractory and high-risk MM hovers around 30%. Importantly, the majority of these patients are still prone to experiencing relapses [3]. Making the treatment even more challenging, assessment indicators such as CR or stringent complete response (sCR) for gauging favorable treatment responses and low-risk disease progression exhibit certain limitations. There is an urgent need to explore highly effective evaluation methods or indicators to assess the extent of MM response accurately. This is essential for precisely determining the treatment effectiveness in patients with MM and establishing a robust foundation for further clinical diagnosis and treatment. Both minimal residual disease (MRD) and serum free light chain ratio (sFLCR) play pivotal roles as diagnostic and therapeutic monitoring indicators for MM. However, they each come with their own set of advantages and disadvantages when evaluating treatment effects. In this paper, we present a comprehensive review of the application and correlation between MRD and sFLCR in the context of MM treatment.

## Application of MRD in the treatment for MM

Over the last decade, there has been consistent improvement in the therapeutic outcomes of MM. This improvement is particularly notable with the continuous integration of new drugs and their combination with transplantation treatments, significantly elevating progression-free survival (PFS) and OS. As the conventional assessment standard of CR is no longer entirely adequate for evaluating patient prognosis, the introduction of MRD as a novel assessment tool becomes crucial. Despite OS being a reliable indicator for clinical purposes, it does have the drawback of being time-consuming. This drawback considerably extends the timeframe from the development to the clinical application of new drugs, posing a challenge to the timely treatment of patients. Particularly, as treatment methods and approaches progressively improve, the PFS and OS for patients with MM are being gradually extended. Relying solely on OS to assess the diagnosis and treatment effectiveness of MM would entail a prolonged demonstration period to showcase the benefits of new drugs. By substituting OS with MRD as the efficacy evaluation standard for MM, the process of demonstrating the effectiveness and advantages of new drugs can be significantly expedited [4]. The primary detection methods for MRD encompass flow cytometry, next-generation sequencing (NGS), and positron emission tomography–computed tomography (PET-CT).

### Main methods for MRD detection

NGS, an advanced high-throughput sequencing technique, possesses a sensitivity level of up to 10–6 and is applicable to over 90% of patients with MM. The fundamental concept of NGS involves sequencing the specific IgH of all plasma cells in the test sample using primers from a primer library and examining their genetic diversity. Bai have demonstrated the remarkable detection capabilities of NGS, suggesting that individuals identified as NGS-MRD-negative may still undergo further remission and extended PFS [5]. However, the existing challenges include the complexity of the current NGS method, the intricacy and time-consuming nature of the operation, limited testing platforms, and the absence of standardized experimental protocol design and authoritative data interpretation, all of which require urgent resolution.

Next-Generation Sequencing (NGS) for the detection of Minimal Residual Disease (MRD) has been demonstrated to have a strong predictive value for both Progression-Free Survival (PFS) and Overall Survival (OS) in patients. This technique offers the advantage of requiring a minimal amount of bone marrow specimen, which facilitates its application, especially for the analysis of specimens that have been preserved for extended periods, thereby supporting retrospective assessments. However, the clinical routine application of NGS for MRD detection is currently hindered by the necessity of having pre-treatment samples to ascertain tumor-specific sequences. This limitation presents a challenge in the widespread implementation of this approach in clinical settings.

Nanni utilizing the high affinity of tumor tissues for the 18F-FDG tracer, PET-CT enables a more precise evaluation of pathological alterations both within and beyond the bone marrow, facilitating the identification of focal osteolytic lesions [6]. In comparison to flow cytometry and NGS, PET-CT is more effective in monitoring extramedullary lesions. Many guidelines include PET-CT among the techniques for MRD detection. However, the reliance of PET-CT on FDG uptake for diagnosis introduces the possibility of false-positive or false-negative results, particularly in the presence of inflammatory or infectious diseases characterized by heightened FDG uptake.

Multi-parameter flow cytometry (MFC) offers notable advantages in MRD detection, including robust adaptability, high sensitivity, and specificity. The concurrent analysis of multiple parameters allows for the monitoring of MRD exhibiting phenotypic changes post-treatment. Additionally, MFC boasts a short detection time and relatively lower costs compared to PET-CT, establishing it as the primary method for MRD detection. The detection sensitivity of the first-generation MFC ranges from 10^–4^ to 10^–5^, while the second generation can achieve a sensitivity of 10^–6^. In a study by Montero et al. [7], a comparison between the two generations revealed that 25% of patients initially classified as MRD-negative by first-generation MFC showed positive MRD results with the second generation, leading to significantly shorter PFS compared to the remaining 75% of patients. Despite its prominence, MFC detection faces certain unresolved issues, including the standardization of detection protocols, such as the selection of schemes and reagent combinations, sample freshness, and the interpretation of results.

### Application of MRD in the evaluation of MM efficacy

The 2016 revision of the International Myeloma Working Group (IMWG) guidelines marked the inclusion of negative MRD in the bone marrow as a key efficacy evaluation criterion. Furthermore, sustaining MRD negativity for over one year was identified as a benchmark indicative of long-term survival and, in certain instances, potential cure. Yang et al. [8] observed that patients who sustained negative MRD for over 12 months experienced notably prolonged PFS and OS. This supports the notion that the duration of maintaining negative MRD can function as a dependable prognostic indicator. Additionally, they noted an extended OS in patients with negative MRD lasting more than 6 months. This underscores the significance of the duration of negative MRD as a potentially crucial prognostic factor that warrants careful consideration. Numerous reports have also emphasized that positive MRD constitutes an independent factor associated with a poor prognosis, necessitating vigilance. According to the research conducted by Kazandjian et al. [9], following autologous hematopoietic stem cell transplantation in patients with MM, the PFS in individuals with MRD-negative status was at least double that of those who were MRD-positive. The study emphasized the significance of MRD-negativity as a prognostic indicator, highlighting its pivotal role in enhancing OS. Consequently, the selection of a treatment regimen that effectively increases the rate of MRD-negativity is crucial. Roshal et al. [10] observed that among a cohort of patients ineligible for transplantation, 30% of those who achieved CR or very good partial response (VGPR) after undergoing six cycles of chemotherapy, demonstrated negative MRD status. This subgroup demonstrated a 3-year PFS rate of 90% and an OS rate of 94%. Importantly, their survival duration was notably higher compared to patients who tested positive for MRD. Yang et al. [8] conducted research on MRD and noted a lack of synchronization between the MRD status and conventional efficacy assessments, such as CR, particularly concerning the timing of these assessments. The prognostic significance of being MRD-negative is essentially equivalent to that of sCR. However, within the study cohort, there was a notably higher percentage of individuals who were MRD-negative (70%) compared to those with sCR (29.5%). This indicates that over 50% of patients achieving MRD negativity exhibit a favorable prognosis, even if they have not reached the level of sCR in terms of efficacy. This suggests that MRD is more sensitive in evaluating the prognosis of MM than currently available clinical efficacy evaluation criteria. In addition, although they compare the changes of multiple indicators in patients with negative and positive MRD, there is a lack of research on the dynamic changes of MRD, which may be a comprehensive clinical efficacy and prognosis evaluation system.

These investigations collectively suggest that MRD holds the potential to supplant PFS as a novel therapeutic endpoint for MM [11–14]. Patients who achieve negative MRD status, particularly those maintaining negativity over an extended period, exhibit significantly lower recurrence rates compared to individuals who are MRD-positive, leading to prolonged PFS and improved OS. These findings provide a dependable foundation for the clinical monitoring and treatment of MM. Patients whose MRD status remains positive should continue their treatment to strive for MRD negativity, aiming for improved efficacy. In cases of rapid relapse post MRD conversion, examinations for extramedullary recurrence, among other factors, are recommended, allowing for an enhanced treatment approach to facilitate future MRD conversions. Patients consistently exhibiting negative MRD should undergo regular monitoring, possibly incorporating combined assessments using PET-CT, second-generation MFC, and NGS to achieve more profound therapeutic effects.

### Issues in clinical application of MRD

Currently, all three primary methods employed for MRD detection face certain unresolved challenges. PET-CT, while effective in monitoring extramedullary lesions, involves a high cost and is prone to some false positives and false negatives. NGS, on the other hand, is hindered by operational complexities, high costs, and time-consuming procedures, posing difficulties in its widespread adoption in clinical practice. Flow cytometry, although widely used, lacks standardized protocols due to variations in measurements among different diagnostic centers in China. This includes discrepancies in the number of cells used for the assay and the selection of antibodies, highlighting the need for well-defined uniform standards in the standardization of flow cytometry for MRD detection [15]. Next are the issues related to sensitivity and the frequency of detection: Should patients with negative MRD undergo regular MRD testing over an extended period? What should be the optimal interval between each test to ensure close disease monitoring while balancing patient interests and conserving medical resources? Addressing technical issues involves enhancing sensitivity to improve the MRD detection rate, potentially leading to more effective disease management for certain patient cohorts [16–19]. However, the sensitivity of MFC detection reported in relevant reports is relatively low, which can easily cause false negatives in some MRD cases. It is necessary to develop detection techniques with higher sensitivity and ease of popularization to better assist in the diagnosis and treatment of MM. Additionally, the localized distribution of bone marrow infiltration in MM poses a challenge, as a negative result may not entirely signify the eradication of the disease. These are collective challenges that require collaborative efforts for resolution. The value of MRD in prognostic evaluation and guiding treatment selection in MM patients has been recognized by scholars both domestically and internationally, but there is still no conclusion on whether MRD can guide the discontinuation of drugs in patients with multiple myeloma (MM).

## Application of sFLCR in MM treatment

Serum free light chain (sFLC) is a highly sensitive marker utilized in the diagnosis of plasma cell diseases. Benson and Sasson etc. finds clinical application in diagnosing, monitoring treatment, and assessing the prognosis of plasma cell diseases, with sFLCR emerging as an independent prognostic indicator for unfavorable outcomes in patients with MM [20–23]. As the calculation of sFLCR involves only polyclonal and sFLC associated with MM pathogenesis, its sensitivity in detecting monoclonals is significantly higher than that of sFLC alone in MM diagnosis and treatment [24].

### Application of sFLCR in the diagnosis and efficacy evaluation of MM

During clonal proliferation of plasma cells, the secretion is predominantly of monoclonal immunoglobulin or monoclonal free light chain molecules with identical genotypes. In patients with MM, the abnormal increase in bone marrow plasma cells results in a significant elevation in the synthesis of monoclonal immunoglobulin or monoclonal free light chain, a phenomenon that intensifies with the progression of the disease. Studies conducted by Li et al. [25] and Huo et al. [26] have affirmed that sFLCR can serve as a valuable complement, providing dependable evidence for the early diagnosis of MM by Lu et al. [27] conducted a study revealing aberrations in sFLC levels or their ratio among patients with MM. Comparatively, the sFLC levels in groups other than ISS stage III patients were notably lower. Following effective combination chemotherapy, there was a significant reduction in sFLCs. However, minimal changes were observed in sFLCs among advanced patients (ineffective group), indicating that combination chemotherapy can effectively decrease tumor burden and the synthesis of free light chains by Jiang et al. [28] discovered that in patients with MM sharing the same immune subtype, those with low sFLCR experienced a more favorable prognosis. In contrast, patients with high sFLCR exhibited higher Durie-Salmon staging system, elevated β2-microglobulin levels, and a significant rise in blood creatinine, indicating more severe renal function impairment compared to those with low sFLCR. Wang et al. [29] conducted a study indicating that, in comparison to patients in the low sFLCR group, those in the high sFLCR group exhibited higher levels of β2-MG, increased plasma cell counts, and more abnormal fluorescence in situ hybridization results at the initial diagnosis. Furthermore, patients in the low sFLCR group were more likely to achieve a VGPR or a more advanced level of response compared to those in the high sFLCR group. This difference may be attributed to the lower tumor load, lower proliferation rate of tumor cells, and reduced invasiveness in the former group. Another study by Qiang et al. [30] demonstrated that sFLCR can serve as a prognostic indicator for newly diagnosed MM and can be used to independently determine the prognosis of MM. Patients in the low sFLCR group exhibited significantly higher 5-year OS than those in the high sFLCR group. However, the patients they selected were mainly treated with 73% bortezomib, so the dynamic value of sFLCR in hematopoietic stem cell transplantation patients needs further in-depth research. Given the valuable role of sFLC and sFLCR in the diagnosis, treatment, and prognosis evaluation of plasma cell diseases [31, 32], they have been incorporated into the IMWG efficacy evaluation criteria.

sFLCR serves as a cost-effective method for the diagnosis and monitoring of MM and has gained widespread use in clinical practice. Among newly diagnosed patients, elevated sFLCR levels indicate a substantial tumor burden and poor treatment response. In contrast to the challenges of quantification and low sensitivity associated with immunofixation electrophoresis, sFLCR offers significant advantages in monitoring the treatment effectiveness of MM. Moreover, the stability of FLC in the serum is notably higher than that of detection methods utilizing urine. According to the conventional efficacy criteria of the IMWG utilized in the Chinese Guidelines for the Diagnosis and Treatment of Multiple Myeloma (Revised in 2020), sFLC alone is adequate for assessing disease changes, and achieving CR requires two consecutive normal sFLCR tests.

### Issues in clinical application of sFLCR

Several studies have highlighted challenges in detecting sFLCR after achieving remission in MM treatment, and the significance of such observations remains unclear. These investigations collectively suggest that there is no statistically significant difference in median PFS between patients in the sCR group and those in the CR group after treatment [33, 34]. Additionally, it is essential to note that in patients with MM with impaired renal function, accurate measurement of sFLC becomes challenging. Since sFLC is predominantly metabolized by the kidneys, with the κ chain degrading faster than the λ chain, abnormal renal function can lead to inconsistencies in the half-lives of κ and λ, resulting in deviations in detection results [35]. Furthermore, the quantitative detection technology for sFLC in China is still underdeveloped, necessitating ongoing and comprehensive research and exploration to lay the groundwork for more extensive clinical applications.

## Correlation between MRD and sFLCR in the treatment of MM

A study by Yang et al. [8] revealed that during the treatment process, the status of MRD and sFLCR may not be synchronized. Approximately 24% of patients exhibited negativity in both MRD and normal sFLCR concurrently, around 18% had MRD negativity before or after the normalization of sFLCR, and roughly 30% of patients with MRD-negative status had abnormal sFLCR. In another study by Deng et al. [19], during a 2-year follow-up, 52% of patients achieved negative MRD before their sFLCR returned to normal, and most patients with negative MRD eventually attained normal sFLCR. However, there were instances of patients presenting with MRD negativity and sFLCR abnormalities, concomitant with extramedullary masses, or experiencing recurrent MRD or disease progression after a period of negative MRD. This suggests that MRD negativity in these cases may be localized, transient, and insufficiently deep. Similarly, among patients with normalized sFLCR, some individuals still had either persistently positive or non-negative MRD. The prognosis for those with normalized sFLCR and persistently negative MRD was significantly better than for those with normalized sFLCR and persistently positive MRD. In a study by Martínez-López et al. [33], among patients who achieved CR, 73% had normalized sFLCR, while the remaining 27% had abnormal sFLCR, with no difference in median PFS between the two groups. Another study by Paiva et al. [36] indicated that patients with negative MRD, detected using 4-color or 8-color flow cytometry, had higher PFS and OS than patients with CR, including those with normalized sFLCR and abnormal sFLCR. Additionally, research by Nadiminti et al. [37] demonstrated that among transplant recipients, those with negative MRD before transplantation exhibited better OS than patients who were MRD-positive.

Chang, Gambella etc. indicated that both MRD positivity and elevated sFLCR serve as independent adverse factors influencing the prognosis of MM [38–43]. Nonetheless, specific case reports have identified noteworthy similarities in prognosis between patients who are MRD-negative and those achieving sCR. Both cohorts have shown significantly superior outcomes compared to patients achieving CR, encompassing those with normalized sFLCR and abnormal sFLCR. Nevertheless, exceptions exist, particularly regarding the duration of MRD negativity. Patients with continuous MRD negativity for over 12 months, for 6 months, and for less than 6 months show varying prognoses. The duration of negative MRD emerges as a crucial factor, with a longer negative MRD duration correlating with a more favorable prognosis. The outcomes are influenced by the selection of the detection method, the timing of the detection, and the depth of the MRD assessment. Several studies have consistently demonstrated that patients with negative MRD detected using 4- to 8-color flow cytometry exhibit superior PFS compared to conventional CR patients or even sCR patients [37–43]. While studies support the utility of MRD and sFLCR as prognostic markers, some research may present contradictory results or limitations. For instance, certain studies might reveal discrepancies between MRD and sFLCR status, suggesting potential challenges or limitations in their clinical applicability. Additionally, conflicting findings regarding the correlation between MRD negativity and improved outcomes, despite abnormal sFLCR, highlight the need for further investigation into the underlying mechanisms and clinical implications. By acknowledging and addressing these conflicting perspectives, we can gain deeper insights into the complexities of MM management and refine treatment strategies accordingly.

## Autophagy in multiple myeloma: implications for bortezomib resistance and minimal residual disease persistence

Autophagy plays a dual role in bortezomib resistance in multiple myeloma (MM), acting as both a survival mechanism and a contributor to treatment resistance. Dysregulated autophagy can promote MM cell survival and diminish the efficacy of bortezomib, while targeting autophagy pathways in combination with bortezomib holds promise for overcoming resistance and improving treatment outcomes. The interplay between autophagy and bortezomib resistance represents a critical area of research with significant implications for enhancing MM therapy efficacy. Autophagy plays a dual role in multiple myeloma (MM) by contributing to both bortezomib resistance and the persistence of minimal residual disease (MRD). Dysregulated autophagy enables MM cells to survive bortezomib-induced stress and adapt to chemotherapy, potentially leading to MRD persistence and disease relapse. Targeting autophagy pathways in combination with bortezomib holds promise for overcoming resistance and eliminating MRD, highlighting the importance of understanding this complex interplay for developing effective therapeutic strategies in MM [44].

## Conclusion

The present review underscores the critical roles of minimal residual disease (MRD) and serum free light chain ratio (sFLCR) in the management and treatment of multiple myeloma (MM). MRD, with its enhanced sensitivity, has emerged as a potent prognostic marker, capable of providing insights into treatment response and disease relapse that surpass traditional measures such as complete response (CR) or stringent complete response (sCR). The integration of MRD negativity as a benchmark for treatment efficacy has been increasingly recognized, with sustained MRD negativity correlating with improved progression-free survival (PFS) and overall survival (OS). However, the utility of MRD is not without its challenges, including the need for standardization across various detection methods and the determination of optimal thresholds and timing for its assessment.

sFLCR, on the other hand, offers a non-invasive and cost-effective approach to diagnose, monitor, and prognosticate MM. Its value in reflecting disease burden and treatment efficacy is well-established, yet the interpretation of sFLCR post-remission and its correlation with MRD status require further clarification. The discrepancies observed between MRD and sFLCR statuses highlight the complexity of MM and suggest that a multifaceted approach to disease monitoring may be necessary.

The interplay between MRD and sFLCR, along with the emerging role of autophagy in bortezomib resistance and MRD persistence, presents a rich area for future research. Targeting autophagy pathways in combination with standard therapies may offer a novel strategy to overcome treatment resistance and eradicate MRD, potentially leading to disease-free remission in MM patients.

As we look to the future, the field must address the current disparities in MRD and sFLCR testing, standardize methodologies, and conduct prospective studies to validate their utility in clinical decision-making. The potential of combining MRD and sFLCR assessments with other biomarkers and imaging techniques should be explored to enhance the precision of MM management. Furthermore, the role of peripheral blood as a diagnostic and monitoring tool, including the assessment of cell-free circulating tumor DNA, circulating plasma cells (CPCs), and M-proteins, warrants investigation, particularly given the limitations of bone marrow biopsies.

In conclusion, while MRD and sFLCR have revolutionized the treatment landscape for MM, their full potential is yet to be realized. Continued research and collaboration are essential to refine these tools, ensuring they become integral components of a personalized approach to MM therapy. As we strive for improved patient outcomes, the pursuit of deeper understanding and innovative applications of MRD and sFLCR will no doubt contribute to the ultimate goal of disease eradication and long-term survival for MM patients.

## Data Availability

The relevant supporting data are available from the author upon request.
